# Temporal dynamics of anxiety phenotypes in a dental pulp injury model

**DOI:** 10.1186/s12990-015-0040-3

**Published:** 2015-06-30

**Authors:** Lin Shang, Tian-Le Xu, Fei Li, Jiansheng Su, Wei-Guang Li

**Affiliations:** Laboratory of Oral Biomedical Science and Translational Medicine, School of Stomatology, Tongji University, Shanghai, 200072 China; Department of Developmental and Behavioral Pediatrics, Shanghai Institute of Pediatric Translational Medicine, Shanghai Children’s Medical Center, Ministry of Education-Shanghai Key Laboratory of Children’s Environmental Health, Shanghai Jiao Tong University School of Medicine, Shanghai, 200129 China; Discipline of Neuroscience and Department of Anatomy, Histology and Embryology, Institute of Medical Sciences, Shanghai Jiao Tong University School of Medicine, 280 South Chongqing Road, Shanghai, 200025 China

**Keywords:** Dental pulp injury, Pain, Anxiety, Social phobia, Synaptic plasticity

## Abstract

**Background:**

Accumulating clinical and preclinical evidence indicates that chronic pain is often comorbid with persistent low mood and anxiety. However, the mechanisms underlying pain-induced anxiety, such as its causality, temporal progression, and relevant neural networks are poorly understood, impeding the development of efficacious therapeutic approaches.

**Results:**

Here, we have identified the sequential emergence of anxiety phenotypes in mice subjected to dental pulp injury (DPI), a prototypical model of orofacial pain that correlates with human toothache. Compared with sham controls, mice subjected to DPI by mechanically exposing the pulp to the oral environment exhibited significant signs of anxiogenic effects, specifically, altered behaviors on the elevated plus maze (EPM), novelty-suppressed feeding (NSF) tests at 1 but not 3 days after the surgery. Notably, at 7 and 14 days, the DPI mice again avoided the open arm, center area, and novelty environment in the EPM, open field, and NSF tests, respectively. In particular, DPI-induced social phobia and increased repetitive grooming did not occur until 14 days after surgery, suggesting that DPI-induced social anxiety requires a long time. Moreover, oral administration of an anti-inflammatory drug, ibuprofen, or an analgesic agent, ProTx-II, which is a selective inhibitor of Na_V_1.7 sodium channels, both significantly alleviated DPI-induced avoidance in mice. Finally, to investigate the underlying central mechanisms, we pharmacologically blocked a popular form of synaptic plasticity with a GluA2-derived peptide, long-term depression, as that treatment significantly prevented the development of anxiety phenotype upon DPI.

**Conclusions:**

Together, these results suggest a temporally progressive causal relationship between orofacial pain and anxiety, calling for more in-depth mechanistic studies on concomitant pain and anxiety disorders.

## Background

Anxiety disorders are a group of mental syndromes characterized by excessively unpleasant feelings of distress or uneasiness caused by fear of the future or dread regarding anticipated events [1]. Anxiety disorders can be categorized into specific phobia, generalized anxiety disorder, obsessive–compulsive disorder, panic disorder, post-traumatic stress disorder, and social anxiety disorder. Clinically, anxiety is an affective disorder that can be comorbid with chronic pain [2, 3]. The two afflictions synergistically affect the quality of life of patients. Preclinically, growing evidence [4] has implicated anxious phenotypes in animal models of chronic pain. These include inflammatory pain, associated with tissue damage or the infiltration of immune cells, and neuropathic pain, associated with damage or abnormal function of the nervous system [5–7]. Despite phenomenological implications of the pain-caused anxiety phenotypes, little is known on mechanisms mediating this re-enforcing interaction between chronic pain and anxiety. Recently, two forms of synaptic plasticity, pre- and post-synaptic long-term potentiation (LTP), in synapses of anterior cingulate cortex (ACC) have been identified to be mechanistically linked to the interaction between chronic pain and anxiety [8]. However, more in-depth studies [9] considering the causality, temporal progression, and neural mechanisms are necessary to further clarify the interaction between pain and anxiety.

As a prevalent type of orofacial pain [10–12], dental pain, such as toothache, produces a severely negative effect on quality of life, including eating disturbances, sleep disruption [13], and mood changes, altering negative affectivity and anxiety vulnerability [10]. The primary cause of toothache is injury to the uniquely innervated dental pulp. Thus, rodent models of this injury (i.e. dental pulp injury, DPI) enable examination of the biological mechanisms of orofacial pain that correlate with human toothache [14, 15]. Mechanical exposure of the dental pulp [12] induces inflammatory changes in the pulp as early as 1 day and periradicular changes 5 days after the procedure. Exposure of dental pulp to the oral environment results in infection and subsequent necrosis of pulp, while a chronic course of exposure further aggravates dental pulp pathology [14] and promotes expression of nociceptive ion channels including Na_V_1.7 [16], Na_V_1.8 [17], class A Ca^2+^ [18], and TRPA1 [19] channels. The growing understanding on the orofacial pain sensation is encouraging, while changes in mood and anxiety levels associated with DPI-induced neuroinflammatory pain [14] remain unexplored, although the development of therapeutic treatments for orofacial pain and the associated affective disorders relies on such research.

In the present study, we used the DPI model to investigate the causality, temporal progression, and potential mechanisms underlying pain-induced anxiety in mice. Based on the histological characterization of dental pulp and behavioral evaluation of daily life activities, including changes in drinking, feeding, body weight, and pain-like behaviors, respectively, we further compared anxiety phenotypes in mice carrying this specific form of chronic pain to sham controls. Through a comprehensive examination of anxious behaviors in DPI mice at different time points after surgery, we established the causality, in a temporally progressive manner, between anxiety and orofacial pain.

## Results

### Histological and functional verification of DPI

**Figure 1:**
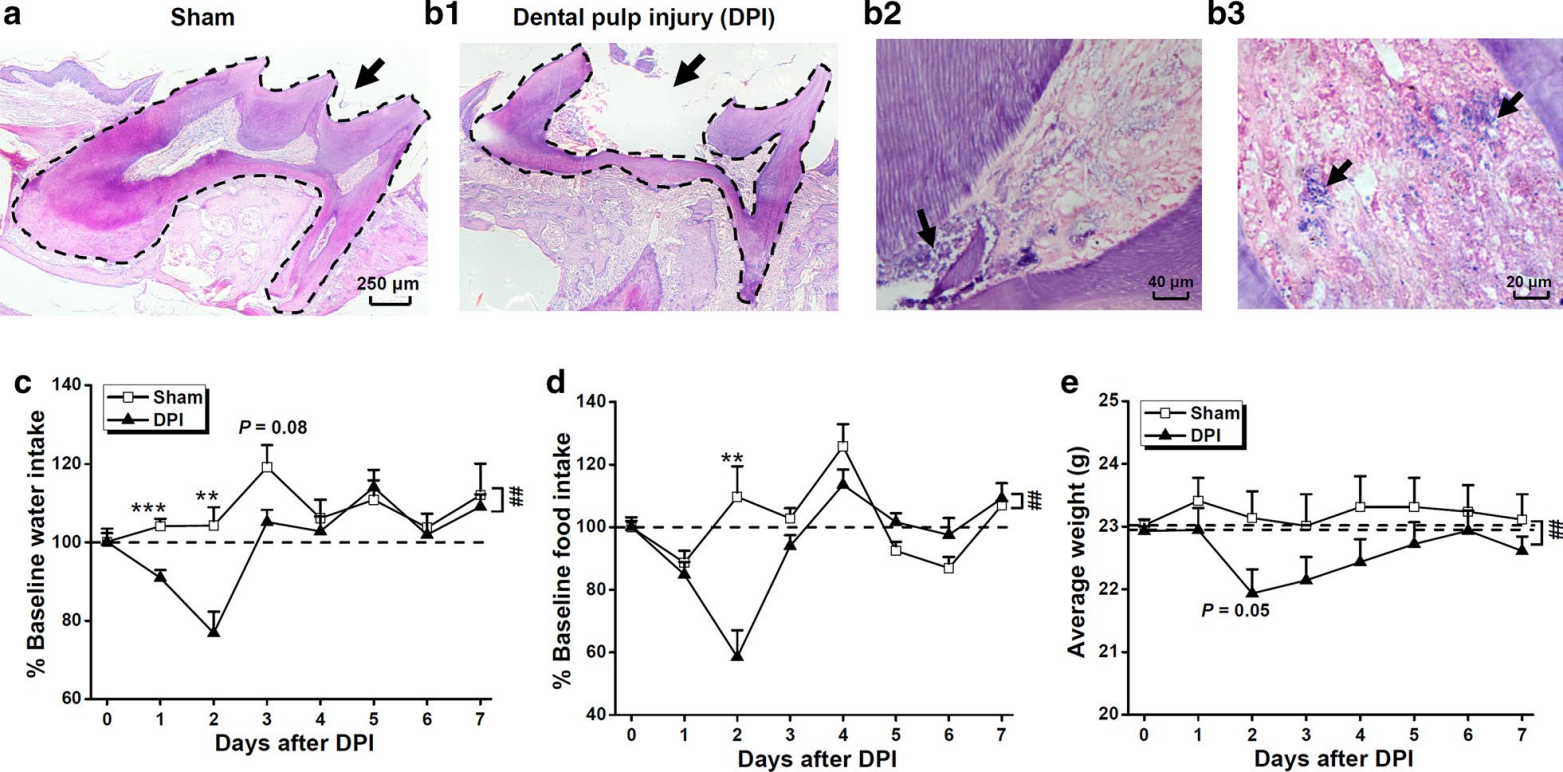
Histological and behavioral characterization of dental pulp injury. **a**, **b** Hematoxylin and eosin (H&E) stain of sham and injured teeth 7 days after experimental surgery illustrating the extent of pulp exposure. The images in (**a**) and (**b1**) were in a comparable scale, and the *dashed lines* indicated the intact (**a**) and injured (**b1**) dental pulps, respectively. **b2** and **b3** are images of DPI in different scales for showing the particular pathological changes. **c**–**e** Quantification of water intake (**c**), food intake (**d**), and bodyweight (**e**) subsequent to sham control or dental pulp injury (DPI) surgery. *Note*: **c** and **d** show values that have been normalized based on the average water and food intake values from the 2 days (*dashed lines*) prior to the surgery, respectively. The *two dashed lines* in (**e**) represent the average bodyweights from the 3 days prior to the sham control and DPI surgeries. All values are shown as mean ± SEM. *n* = 8 and 9 mice for the sham and DPI groups, respectively. **P < 0.01, ***P < 0.001, sham vs. DPI, unpaired Student’s *t* test. A two-way ANOVA revealed significant differences between the sham and DPI groups in terms of water (*P* = 0.001) and food (*P* = 0.007) intake as well as body weight (*P* = 0.001). Please *see the text* for more details.

We first verified the efficacy of our surgical procedure in establishing DPI by performing histological analyses and behavioral characterizations of feeding-related activities [13, 14]. Gross histological changes were assessed by examining hematoxylin and eosin (H&E)-stained slide-mounted cryosections of decalcified maxillae. Specifically, we looked for successful degradation of the coronal pulp of the left maxillary first molar (see “Methods”) 7 days after the DPI procedure (Figure 1b1) in the experimental mice but not the sham controls (Figure 1a). We found in the DPI animals that the radicular part of the injured pulp was partly reserved (Figure 1b1) but necrotic (Figure 1b2). Notably, a significant infiltration of blue staining-characteristic inflammatory cells such as neutrophils, lymphocytes, and monocytes in the remaining pulpal tissues occurred in the DPI (Figure 1b3) but not the shame control (data not shown) animals. These morphological results verified the composite inflammatory and neuropathic mechanisms underlying DPI-caused damage [14]. Overall, our observation on the changes in the dental tissue matched the pathological development of DPI reported previously [13], shown that the injured dental pulp progressively advanced from vital to partially degraded status.

To further validate the functional consequences of DPI in experimental animals compared with sham controls, we performed an additional examination of feeding activities after surgery. Consistent with previous reports [13, 15], we confirmed the following behavioral changes in our DPI mice. Compared with average daily baseline behaviors (dashed line in Figure 1c, d) before DPI, and to control manipulations, we observed a large decrease in drinking (Figure 1c) and feeding behaviors in the DPI animals (Figure 1d). This effect was significantly smaller or completely absent in control animals, in which the effect subsided within 3 days following the anesthesia and manipulation (Figure 1c, d). A two-way analysis of variance (ANOVA) conducted on water intake throughout the 7 postoperative days revealed a significant difference between the sham control and DPI groups and among different test days [treatment, F_(1,118)_ = 12.070, *P* = 0.001; test day, F_(6,118)_ = 6.419, *P* < 0.001; interaction, F_(6,118)_ = 2.579, *P* = 0.023]. A *t* test revealed a significant difference in water consumption between the DPI and control groups on the first (*P* < 0.001, sham vs. DPI) and second (*P* < 0.01, sham vs. DPI) days, but not on other days. Similarly, a two-way ANOVA conducted on food intake revealed a significant difference between the sham control and DPI groups, as well as over different test days subsequent to the surgery [treatment, F_(1,118)_ = 7.666, *P* = 0.007; test day, F_(6,118)_ = 11.315, *P* < 0.001; interaction, F_(6,118)_ = 8.073, *P* < 0.001], while a *t* test indicated that there was a significant difference between the DPI and control groups on the second day (*P* < 0.01, sham vs. DPI). Echoing these variations in feeding and drinking, the DPI animals exhibited a significantly greater loss in body weight compared with the sham controls, with the largest difference occurring 2 days after injury (Figure 1e). A two-way ANOVA conducted on bodyweight throughout the 7 postoperative days revealed a significant difference between the sham control and DPI groups [treatment, F_(1,118)_ = 10.824, *P* = 0.001; test day, F_(6,118)_ = 0.784, *P* = 0.584; interaction, F_(6,118)_ = 0.313, *P* = 0.929], while a *t* test showed a marginally significant difference between the DPI and control groups on the second day post-surgery (*P* = 0.05, sham vs. DPI). Notably, all of the feeding-related changes had been fully restored to baseline levels by 1 week after the DPI or control manipulation, implying that the effects of the anesthesia and pulp injury on the global physical status of the mice was transient [13]. Together, we considered the DPI model in mice to have been successfully established without causing unintended harm to extraneous body systems.

### Behavioral evaluation of nociception temporally subject to DPI

**Figure 2:**
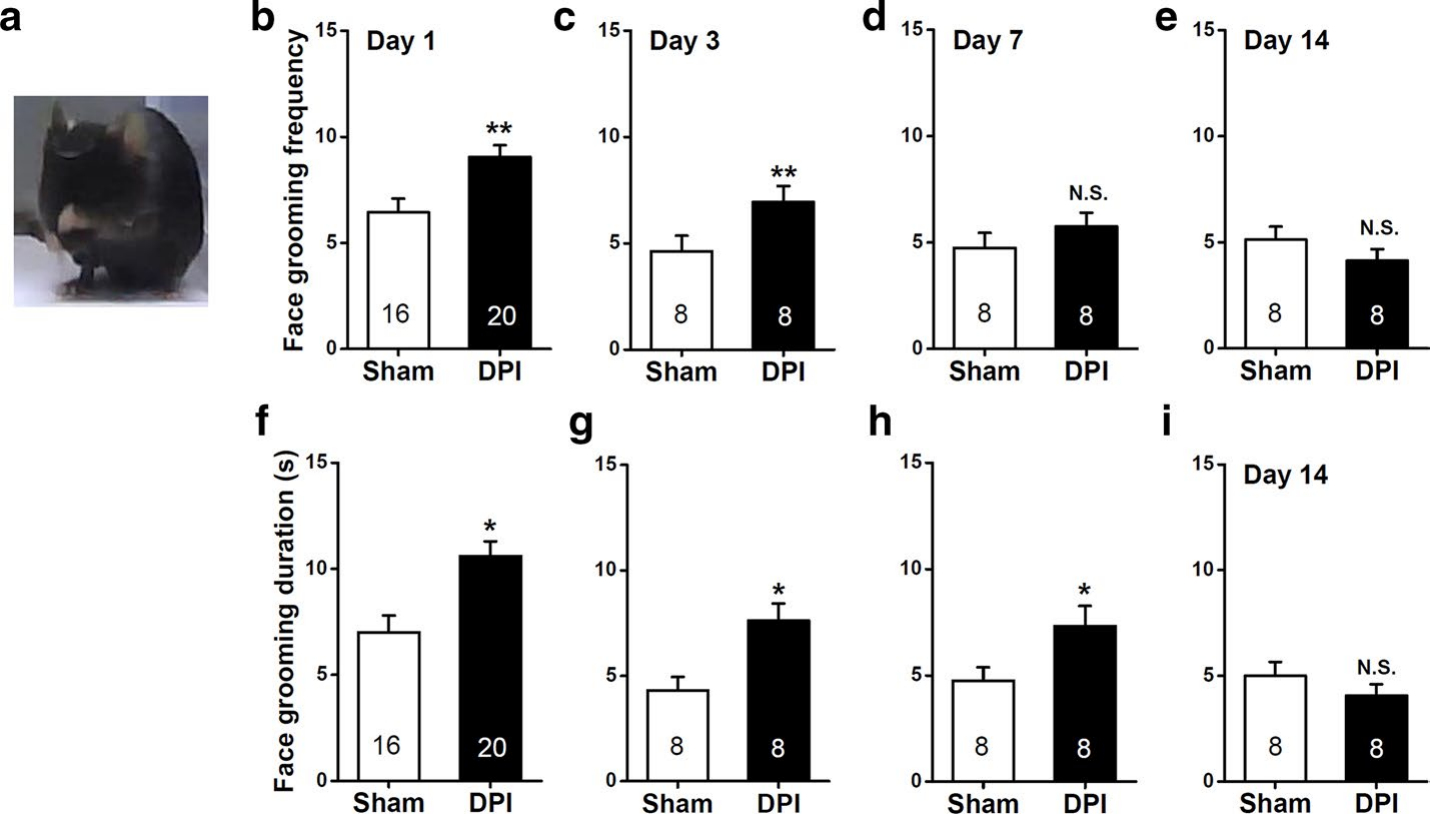
Measurements of pain-like behavior by quantification of face grooming after sham or DPI surgery. **a** An example image showing face grooming of the mice. **b**–**i** The *bar summary* compares the frequency (**b**–**e**) and duration (**f**–**i**) of face grooming during the 30 min test between the sham and DPI mice. All values are expressed as mean ± SEM. *n* = 8–20 mice for each group shown in the figure. *P < 0.05, **P < 0.01, *N.S.* non-significant difference, sham vs. DPI, unpaired Student’s *t* test.

To establish the time-dependent pain-like phenotypes subject to DPI, we performed careful examination on behavioral responses following the sham control and DPI treatment by quantifying the frequency and duration of mice face grooming (Figure 2a), that probably correlates with the nociception changes [15, 20]. On days 1 and 3, DPI mice displayed a significant increase in frequency (day 1, *P* < 0.01, Figure 2b; day 3, *P* < 0.01, sham vs. DPI, Figure 2c) and time spent (day 1, *P* < 0.05, sham vs. DPI, Figure 2f; day 3, *P* < 0.05, sham vs. DPI, Figure 2g) in face grooming compared with sham control. On day 7 after surgery, DPI mice showed significant increase in the duration (*P* < 0.05, sham vs. DPI, Figure 2h) but not frequency (*P* > 0.05, sham vs. DPI, Figure 2d) of face grooming. In contrast, we found no significant differences in the frequency (Figure 2e) and duration (Figure 2i) of face grooming between the sham and DPI mice on day 14. Collectively, these data implicate a gradually decreasing pain-like behavior subject to DPI, which is consistent with the clinical observation associated with dental pain in pulpitis.

### Bell-shaped temporal progression of anxiety subsequent to DPI

**Figure 3:**
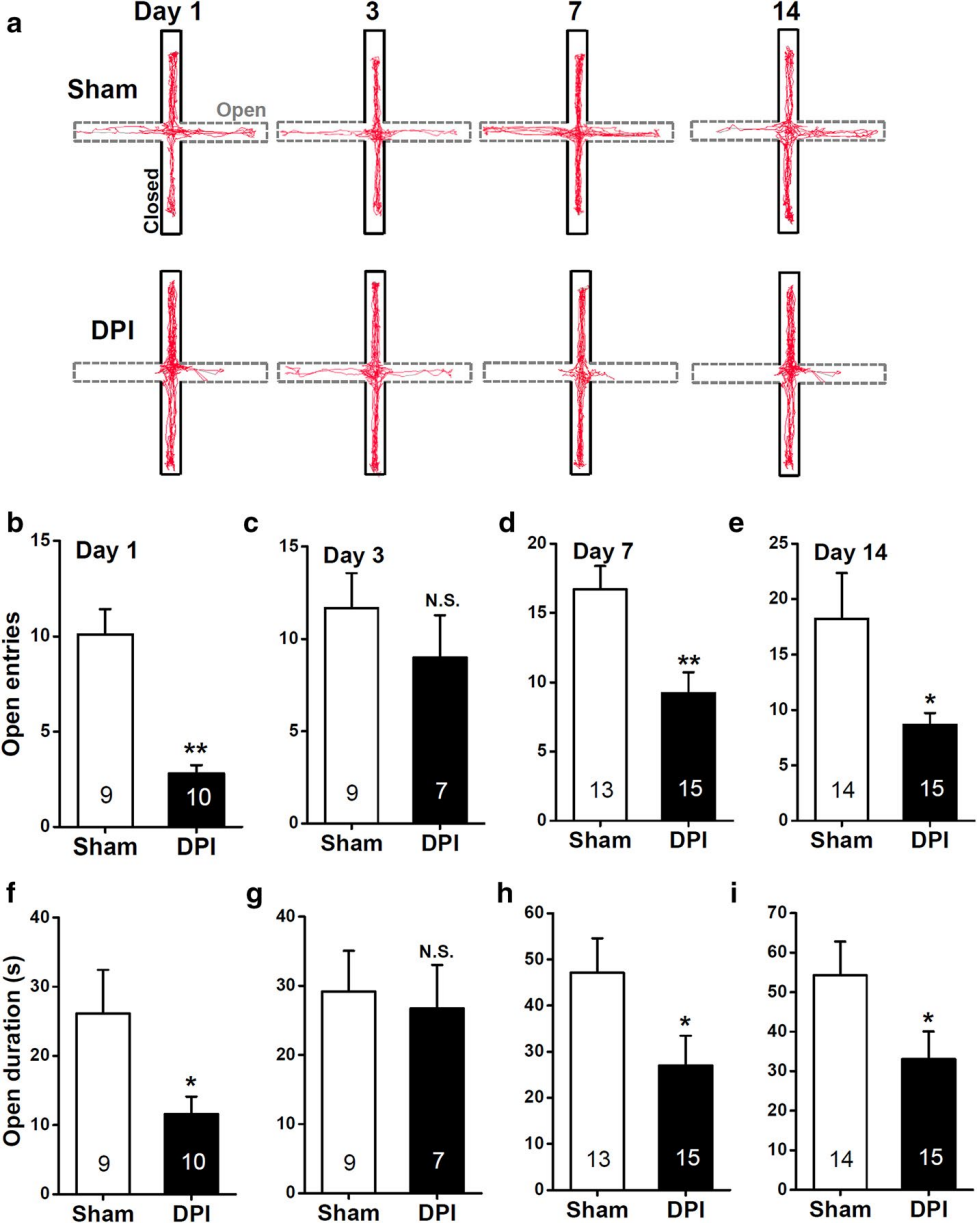
Measurements of anxiety-like behavior in the EPM after sham or DPI surgery. **a** Computer-generated exploration paths of representative sham and DPI mice in the EPM test. Open, *open arms* (*dashed line*, *grey*); closed, *closed arms* (*black*). **b**–**i** The bar summary compares the number of entries (**b**–**e**) and the amount of time spent (**f**–**i**) in the *open arms* between the sham and DPI mice. All values are expressed as mean ± SEM. *n* = 7–15 mice for each group shown in the figure. *P < 0.05, **P < 0.01, *N.S.* non-significant difference, sham vs. DPI, unpaired Student’s *t* test.

To investigate the affective phenotype associated with DPI, we assessed innate anxiety behaviors using the elevated plus maze (EPM) test [21–23] on different days (i.e. days 1, 3, 7, and 14) after the surgery. To reduce the potential influence of confounding habituation effects caused by repeated testing, we produced and tested separate groups of mice at each time point. On day 1, DPI mice displayed a significant decrease (Figure 3a) in entries (*P* < 0.01, sham vs. DPI, Figure 3b) and time spent (*P* < 0.05, sham vs. DPI, Figure 3f) in the open arms of the maze compared with sham mice. In addition, the DPI procedure did not appear to affect general locomotor activity, as indexed by the total distance moved during the EPM tests (distance traveled in 5 min, sham: 13.8 ± 0.9 m, DPI: 11.9 ± 1.5 m; n = 9–10 for each group; sham vs. DPI, *P* > 0.05, sham vs. DPI, unpaired Student’s *t* test, data not shown). Hence, DPI mice appeared to exhibit a genuine increase in anxiety-like behavior in the absence of confounding effects related to possible changes in basal locomotor activity. We speculated that the DPI-induced anxiety observed on the 1st day was probably associated with acute injury per se, also reminiscent of the observed pain-like behaviors shown before (Figure 2b, f). Strikingly, on the 3rd day, DPI mice displayed a similar level (Figure 3a) of entries (*P* > 0.05, sham vs. DPI, Figure 3c) and time spent (*P* > 0.05, sham vs. DPI, Figure 3g) in the open arms of the maze compared with sham mice. These results imply that the anxious phenotype associated with DPI has temporally specific characteristics.

On days 7 and 14 (Figure 3a) after surgery, DPI mice once again displayed increased avoidance to the open arms in the EPM test. Specifically, we found a significant decrease in entries (day 7, *P* < 0.01, sham vs. DPI, Figure 3d; day 14, *P* < 0.05, sham vs. DPI, Figure 3e) and time spent (day 7, *P* < 0.05, sham vs. DPI, Figure 3h; day 14, *P* < 0.05, sham vs. DPI, Figure 3i) in the open arms in the DPI compared with the sham mice. These data implied a bell-shaped temporal progression of the anxiety phenotype, subsequent to the DPI procedure.

### Ethological measurements of DPI-induced anxiety during the EPM test

**Figure 4:**
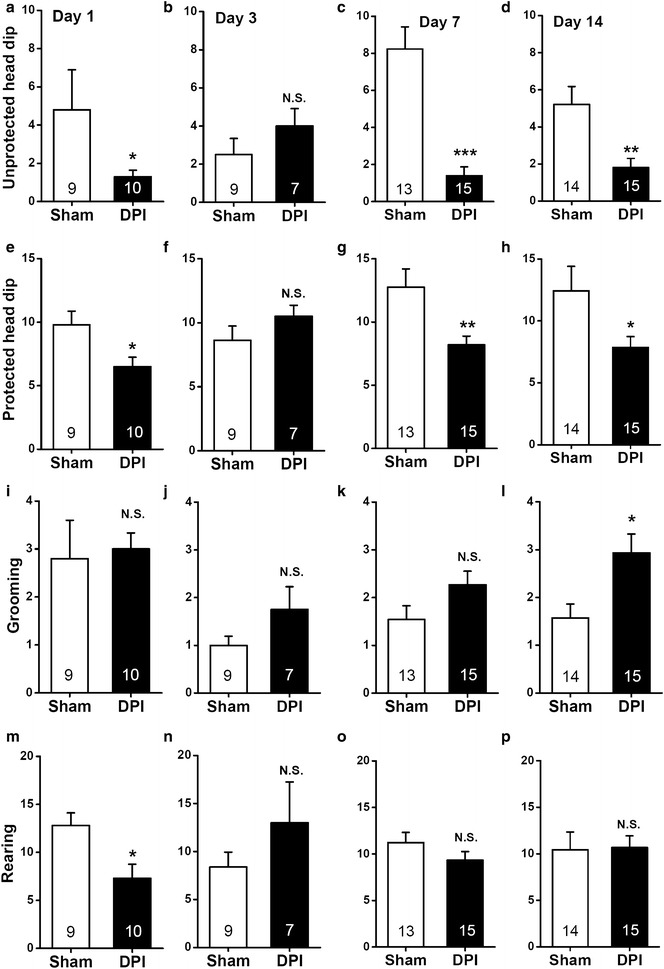
Effects of DPI on ethological measurements taken during the EPM test. **a**–**d** Frequency of unprotected head dips that occurred in the *open arms*; **e**–**h** frequency of protected head dips that occurred in the central area and *closed arms*; **i**–**l** grooming; **m**–**p** rearing. All values are expressed as mean ± SEM. *n* = 7–15 mice for each group shown in the figure. *P < 0.05, **P < 0.01, ***P < 0.001, *N.S.* non-significant difference, sham vs. DPI, unpaired Student’s *t* test.

Figure 4 shows the effects of DPI on quantifiable ethological parameters during the EPM test. Consistent with the DPI-induced changes in entries and time spent in the open arms of the maze shown in Figure 3, DPI significantly decreased the numbers of both unprotected (day 1, *P* < 0.05, Figure 4a; day 3, *P* > 0.05, Figure 4b; day 7, *P* < 0.001, Figure 4c; day 14, *P* < 0.05, sham vs. DPI, Figure 4d) and protected (day 1, *P* < 0.05, Figure 4e; day 3, *P* > 0.05, Figure 4f; day 7, *P* < 0.01, Figure 4g; day 14, *P* < 0.05, sham vs. DPI, Figure 4h) head dips compared with the sham controls on days 1, 7, and 14, but not day 3, respectively. In contrast, we found a significant difference on the frequency of rearing between the sham and DPI mice on day 1 (*P* < 0.05, sham vs. DPI, Figure 4m), but not other time points (all *P* > 0.05, sham vs. DPI, Figure 4n–p). As rearing behavior is correlated with exploration [24], the rearing frequency of DPI mice in most time points (except day 1) was similar to that of the sham controls suggests that the DPI animals exhibited normal exploration behavior. This reinforces the specificity of anxious phenotypes elicited by DPI. Interestingly, a DPI-induced increase in self-grooming emerged by 14 days (*P* < 0.05, sham vs. DPI, Figure 4l), but not other time points (all *P* > 0.05, sham vs. DPI, Figure 4i–k) after the surgery. The significant difference between sham control and DPI mice in terms of self-grooming behaviors models the distinctive phenotype of obsessive–compulsive disorder [25]. In addition, this provides further evidence for the temporal progression of DPI-induced emergence of anxious phenotypes.

### DPI-induced anxiety behavior in the open field test

**Figure 5:**
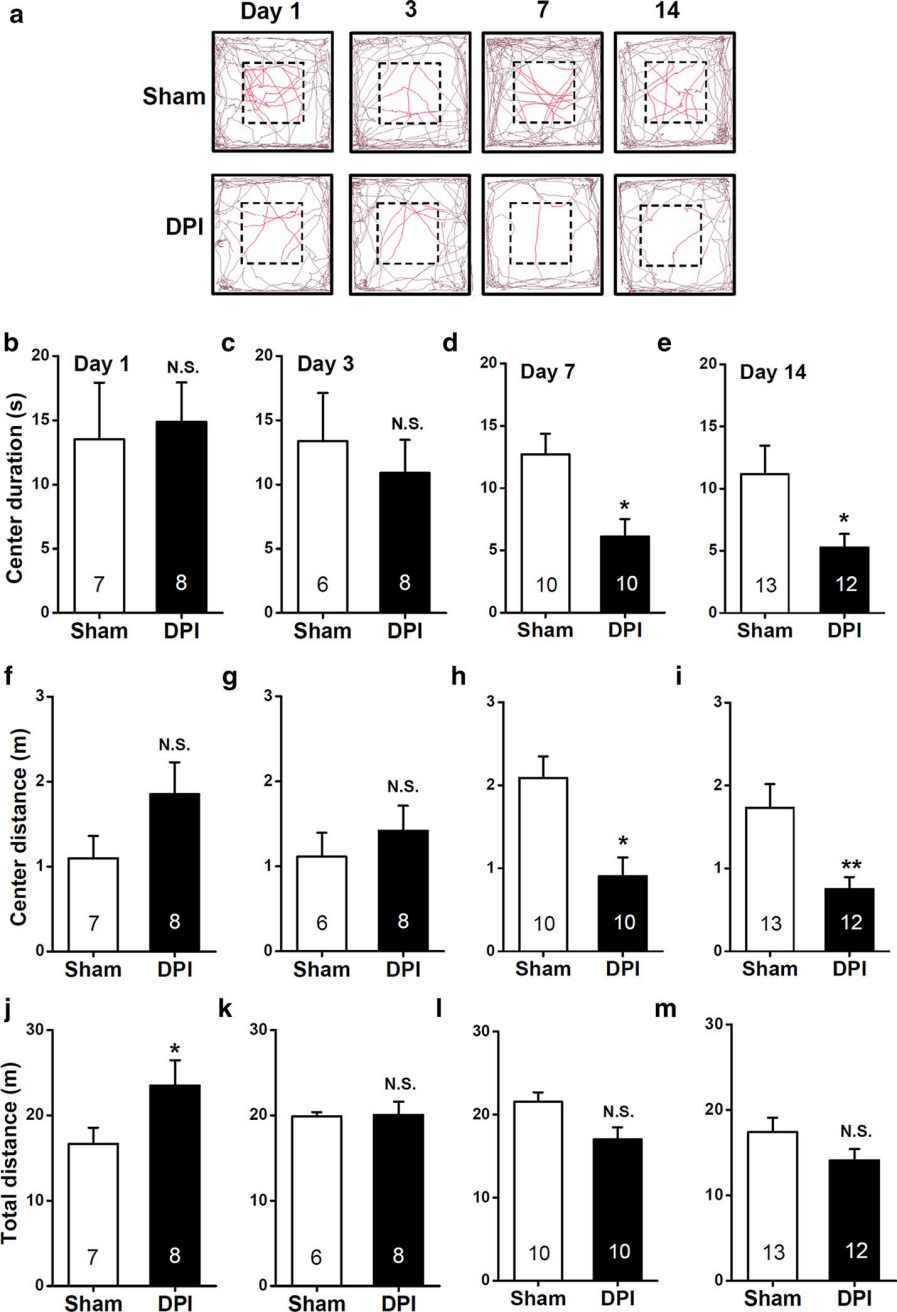
Effects of DPI on behavior in the open field test. **a** Computer-generated exploration paths of representative sham and DPI mice in the open field test. **b**–**m** The *bar summary* compares the time spent (**b**–**e**) and distance traveled (**f**–**i**) in the center area, in addition to the total distance traveled in the entire testing arena (**j**–**m**) between the sham and DPI mice. *P < 0.05, **P < 0.01, *N.S.* non-significant difference, sham vs. DPI, unpaired Student’s *t* test.

Consistent with the above results, the behavioral indices of innate anxiety associated with DPI were further evaluated in the open field test [26]. As shown in Figure 5a, on days 1 and 3, DPI mice showed insignificant difference in time spent (day 1, *P* > 0.05, Figure 5b; day 3, *P* > 0.05, sham vs. DPI, Figure 5f) and distance travelled in the center zone (day 1, *P* > 0.05, Figure 5c; day 3, *P* > 0.05, sham vs. DPI, Figure 5g) compared with that of sham controls. Besides, on day 1 but not 3, DPI increased the total distance moved in the entire open field arena (day 1, *P* < 0.05, Figure 5j; day 14, *P* > 0.05, sham vs. DPI, Figure 5k), arguing for an altered basal activity in the open field. Of note, on days 7 and 14, as with the increase in the behavioral indices of innate anxiety in the EPM test, DPI mice displayed a significant decrease (Figure 5a) in time spent (day 7, *P* < 0.05, Figure 5d; day 14, *P* < 0.05, sham vs. DPI, Figure 5e) and distance travelled in the center zone (day 7, *P* < 0.05, Figure 5h; day 14, *P* < 0.01, sham vs. DPI, Figure 5i) compared with sham controls. DPI did not affect the total distance moved in the entire open field arena (day 7, *P* > 0.05, Figure 5l; day 14, *P* > 0.05, sham vs. DPI, Figure 5m). Thus, DPI appears to have increased the presence of anxious phenotypes over time, as revealed by the mice behavior in the open field test.

### DPI-induced anxiety behavior in the novelty-suppressed feeding test

**Figure 6:**
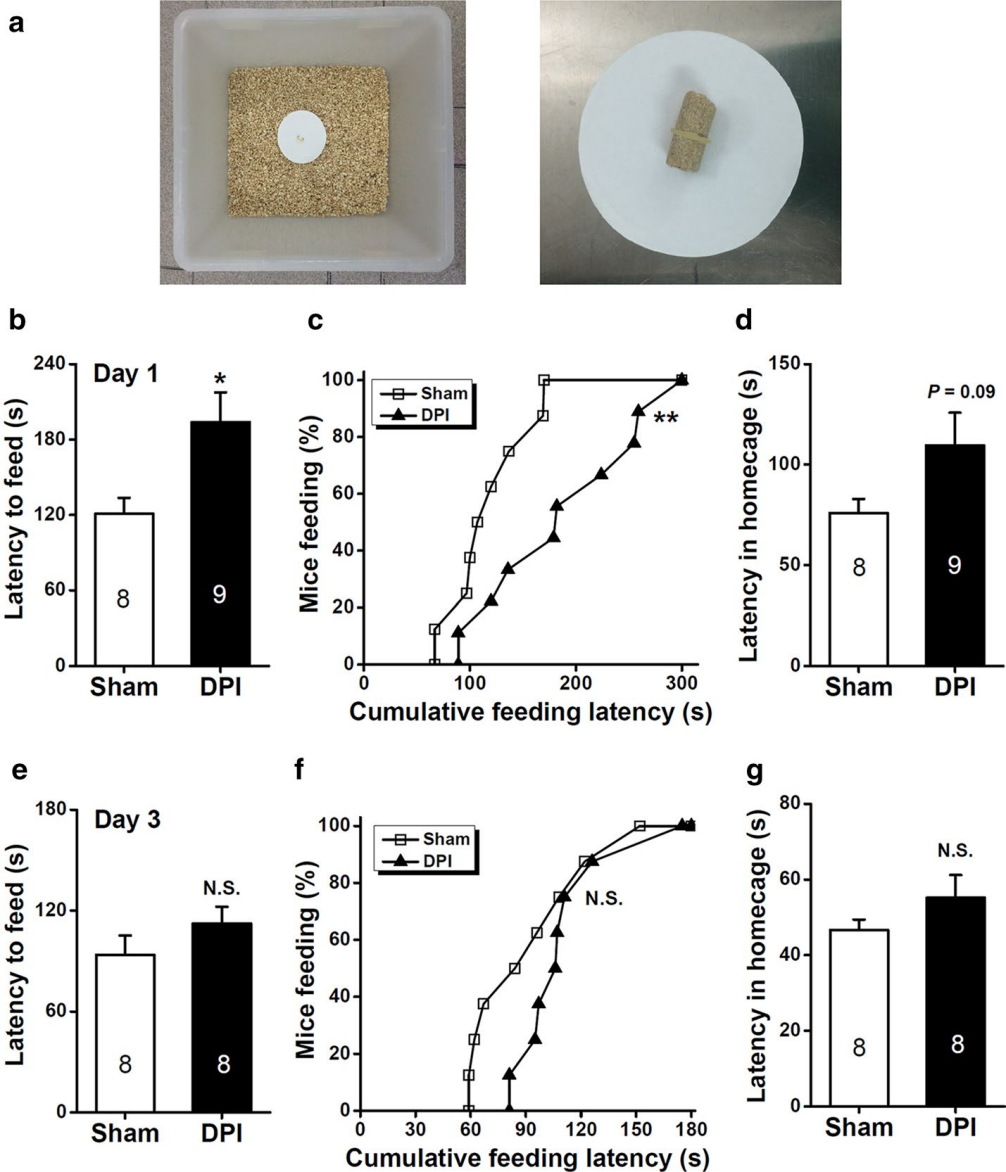
Effects of DPI on behavior in the novelty-suppressed feeding test on days 1 and 3 after surgery. **a** Images show the testing arena and food platform used in the novelty-suppressed feeding test. **b**, **e** The *bar summary* shows the feeding latency for the food on the novel platform. *n* = 8–9 mice for each group shown in the figure. *P < 0.05, *N.S.* non-significant difference, sham vs. DPI, unpaired Student’s *t* test. **c**, **f** Cumulative curves showing the effect of DPI on the distribution of feeding latency for food on the novel platform. *N.S.* non-significant difference, **P < 0.01, sham vs. DPI, one-sample Kolmogorov–Smirnov test. **d**, **g** The *bar summary* shows the feeding latency for the food in the home cage. *n* = 8–9 mice for each group shown in the figure. *N.S.* not significant difference, sham vs. DPI, unpaired Student’s *t* test.

**Figure 7:**
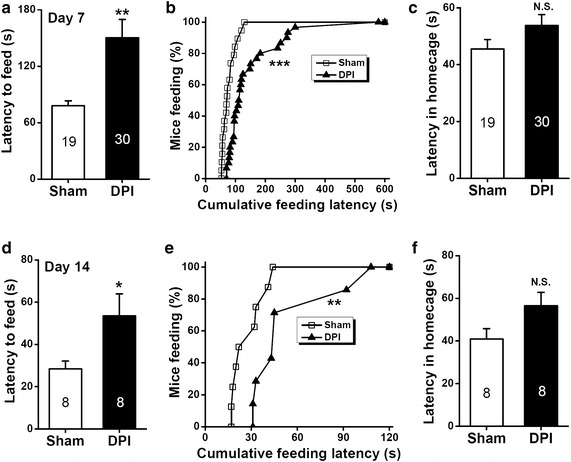
Effects of DPI on behavior in the novelty-suppressed feeding test on days 7 and 14 after surgery. **a**, **d** The *bar summary* shows the feeding latency for the food on the novel platform. *n* = 8–30 mice for each group shown in the figure. *P < 0.05, **P < 0.01, sham vs. DPI, unpaired Student’s *t* test. **b**, **e** Cumulative curves showing the effect of DPI on the distribution of feeding latency for food on the novel platform. **P < 0.01, ***P < 0.001, sham vs. DPI, one-sample Kolmogorov–Smirnov test. **c**, **f** The *bar summary* shows the feeding latency for the food in the home cage. *n* = 8–30 mice for each group shown in the figure. *N.S.* not significant difference, sham vs. DPI, unpaired Student’s *t* test.

The novelty-suppressed feeding (NSF) test provided additional evidence for the notion that DPI increased behavioral indices of innate anxiety [27, 28]. In this test, food-deprived mice were introduced to a novel cage that was larger than their home cage. The novel cage contained a food pellet at its center (Figure 6a). We recorded the latency to feeding onset. Consistent with the time-dependent emergence of anxious phenotype subject to DPI shown in the above behavioral paradigms, DPI significantly increased the feeding latency in the novel environment on days 1 (*P* < 0.05, sham vs. DPI, Figure 6b) but not 3 (*P* > 0.05, sham vs. DPI, Figure 6e), again increased the latency on days 7 (*P* < 0.01, sham vs. DPI, Figure 7a) and 14 (*P* < 0.05, sham vs. DPI, Figure 7d). Moreover, a cumulative distribution analysis of feeding in the novel environment (day 1, *P* < 0.01, sham vs. DPI, Figure 6c; day 3, *P* = 0.159, Figure 6f; day 7, *P* < 0.001, Figure 7b; day 14, *P* < 0.01, sham vs. DPI, Figure 7e) confirmed the time-dependent appearance of anxiety on DPI. In contrast, when we assessed feeding latency in the home cage, the sham control and DPI mice showed overall insignificant average feeding latencies (day 1, *P* = 0.09, Figure 6d; day 3, *P* > 0.05, Figure 6g; day 7, *P* > 0.05, Figure 7c; day 14, *P* > 0.05, sham vs. DPI, Figure 7f). Collectively, the results of multiple behavioral tests indicate that DPI indeed induced a temporally-specific anxiogenic effects after surgery.

### DPI-induced suppression of social exploration emerges more slowly

**Figure 8:**
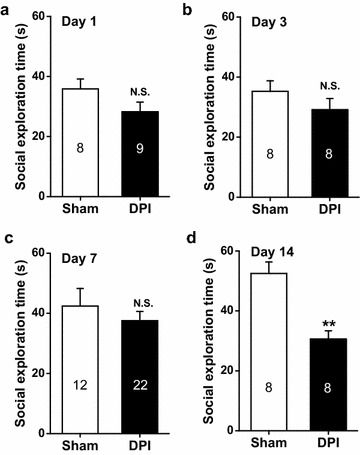
Quantification of time spent engaged in social exploration for sham control and DPI mice at different days following surgery. **a**–**d** represent the results on days 1, 3, 7 and 14, respectively. *n* = 8–22 mice for each group shown in the figure. **P < 0.01, *N.S.* non-significant difference, sham vs. DPI, unpaired Student’s *t* test.

We used a social exploring paradigm to evaluate the social anxiety status [29] of sham control and DPI mice over time. The mean time spent investigating juvenile cohorts is shown in Figure 8. On day 14, but not days 1, 3, nor 7, DPI mice spent a significantly shorter time on exploring an intruder (day 1, *P* > 0.05, Figure 8a; day 3, *P* > 0.05, Figure 8b; day 7, *P* > 0.05, Figure 8c; day 14, *P* < 0.01, sham vs. DPI, Figure 8d) compared with sham control mice, indicative of an increased social phobia at that time (i.e. 14 days). This time-dependent social withdrawal was synchronized with the observed increase in repetitive self-grooming behaviors seen during EPM tests 14 days, but not less days after DPI (Figure 4i–l). These time-dependent changes are reminiscent of a recent study [30] in which an amygdala subregion was found to mediate antagonistic control of social versus repetitive self-grooming behaviors. It is possible that the DPI affect the balanced interaction of separable amygdala neuronal subsets controlling social and repetitive self-grooming behaviors, thus influencing social exploration in a temporally dependent way.

### Anti-inflammatory or analgesia treatment attenuates DPI-induced anxiety

**Figure 9:**
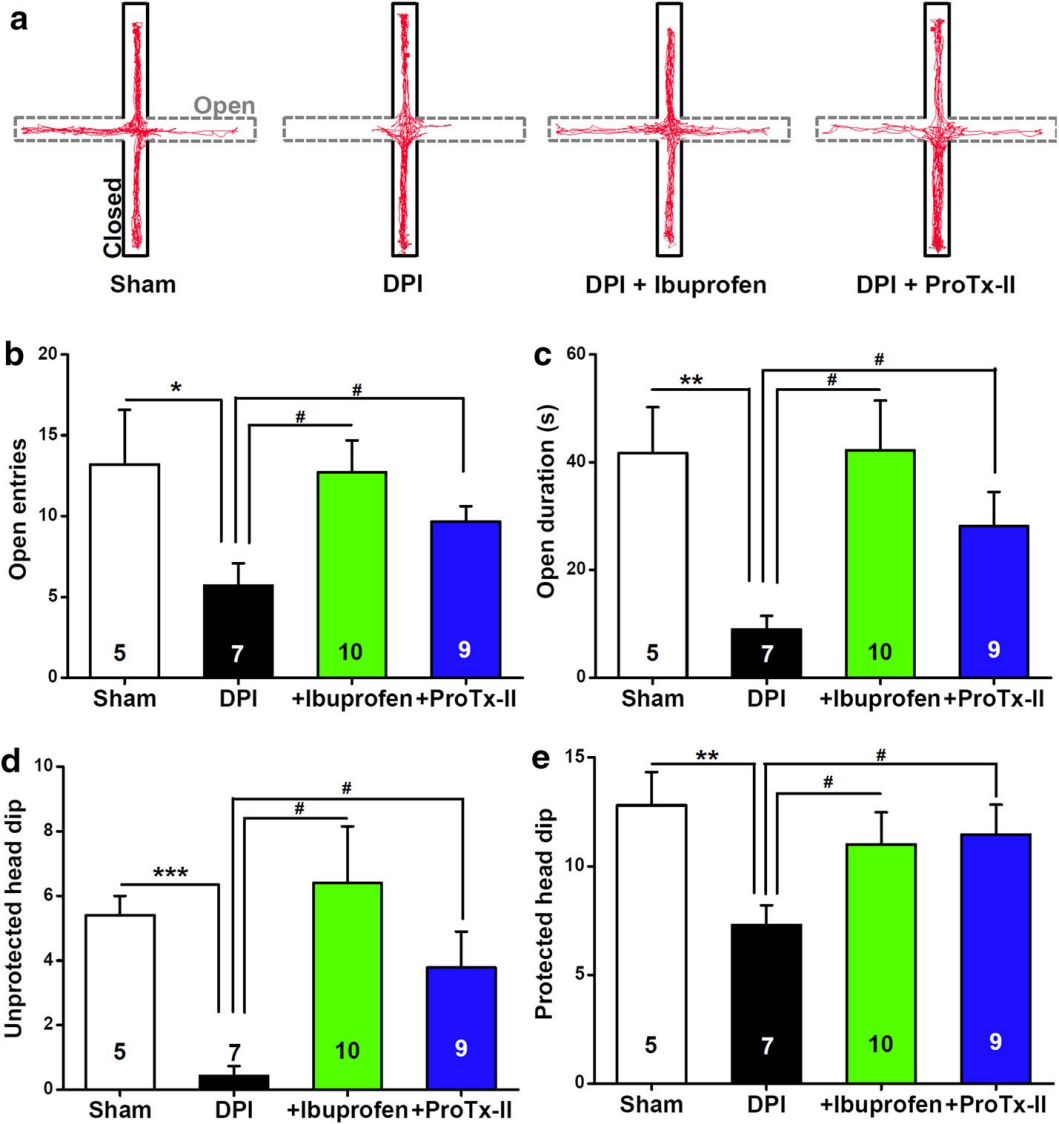
Effects of administration of ibuprofen or ProTx-II on DPI-induced anxiety behaviors in the EPM 7 days after surgery. **a** Computer-generated exploration paths of representative mice in the EPM test subjected to the following conditions: sham control, DPI, DPI plus ibuprofen administration, DPI plus ProTx-II administration. Open, *open arms* (*dashed line*, *grey*); closed, *closed arms* (*black*). **b**, **c** The bar summary shows the number of entries (**b**) and the time spent (**c**) in the *open arm*. **d**, **e** The *bar summary* shows the number of unprotected (**d**) and protected (**e**) head dips during the EPM test. All values are expressed as mean ± SEM. *n* = 5–10 mice for each group shown in the figure. *P < 0.05, **P < 0.01, ***P < 0.01, sham vs. DPI, unpaired Student’s *t* test. ^#^ *P* < 0.05, DPI vs. DPI + ibuprofen or DPI vs. DPI + ProTx-II as indicated, unpaired Student’s *t* test.

To probe the potential cause of DPI-induced anxiety, we administered an anti-inflammatory treatment via oral administration of ibuprofen [31] immediately following the surgery until the day on which the behavioral tests were conducted. By day 7 after DPI, when significant anxiety phenotypes had emerged, such as a strong avoidance to the open arm in the EPM test (Figures 3, 4), administration of ibuprofen significantly reversed the anxious phenotypes in DPI mice (Figure 9a). Specifically, we observed a significant increase in the number of entries (*P* < 0.05, sham vs. DPI; *P* < 0.05, DPI vs. DPI + ibuprofen, Figure 9b) and time (*P* < 0.01, sham vs. DPI; *P* < 0.05, DPI vs. DPI + ibuprofen, Figure 9c) spent in the open arms of the maze compared with DPI mice that did not receive ibuprofen. Moreover, ibuprofen significantly increased the numbers of both unprotected (*P* < 0.001, sham vs. DPI; *P* < 0.05, DPI vs. DPI + ibuprofen, Figure 9d) and protected (*P* < 0.01, sham vs. DPI; *P* < 0.05, DPI vs. DPI + ibuprofen, Figure 9e) head dips during the EPM test, indicating that anti-inflammatory treatment has an anxiolytic effect on DPI-induced anxiety. The present results support the major participation of an inflammation process in DPI pathology over time. Like hyperalgesia with other inflammatory pain models caused by injection of complete Freund’s adjuvant to the hind paw of mice, dental inflammation in DPI (Figure 1b3) may contribute to the progressive development of anxiety phenotypes by promoting orofacial pain.

To further address the role of hyperalgesia in the development of anxiety in DPI, we used ProTx-II, which is a selective Na_V_1.7 channel blocker [32, 33] that presumably produces an analgesic effect by attenuating nociceptive transmission and abnormal excitability of the exposed afferent nerve that innervates the injured pulp in DPI mice. As expected, like ibuprofen, ProTx-II substantially alleviated DPI-induced avoidance to the open maze arm during EPM tests (Figure 9a). Oral administration of ProTx-II produced a significant increase in entries (*P* < 0.05, DPI vs. DPI + ProTx-II, Figure 9b) and time (*P* < 0.05, DPI vs. DPI + ProTx-II, Figure 9c) spent in the open arms compared with untreated DPI mice. In addition, ProTx-II treatment significantly increased the numbers of both unprotected (*P* < 0.05, DPI vs. DPI + ProTx-II, Figure 9d) and protected (*P* < 0.05, DPI vs. DPI + ProTx-II, Figure 9e) head dips during the EPM test, again supporting the anxiolytic effects of analgesia treatment on DPI-induced anxiety. These anxiolytic effects of ProTx-II are reminiscent of a previous study showing the Na_V_1.7 upregulation in painful human dental pulp and burning mouth syndrome [16], and also strengthen the benefits of targeting this channel for antianxiety in addition to the established effects of pain and itch relief [34]. Together, these results collectively established a temporally progressive and manipulatively sensitive causality between DPI pathophysiology (i.e. both inflammation and orofacial hyperalgesia) and anxiety phenotypes.

### Pharmacological blockade of long-term depression reduces DPI-induced anxiety

**Figure 10:**
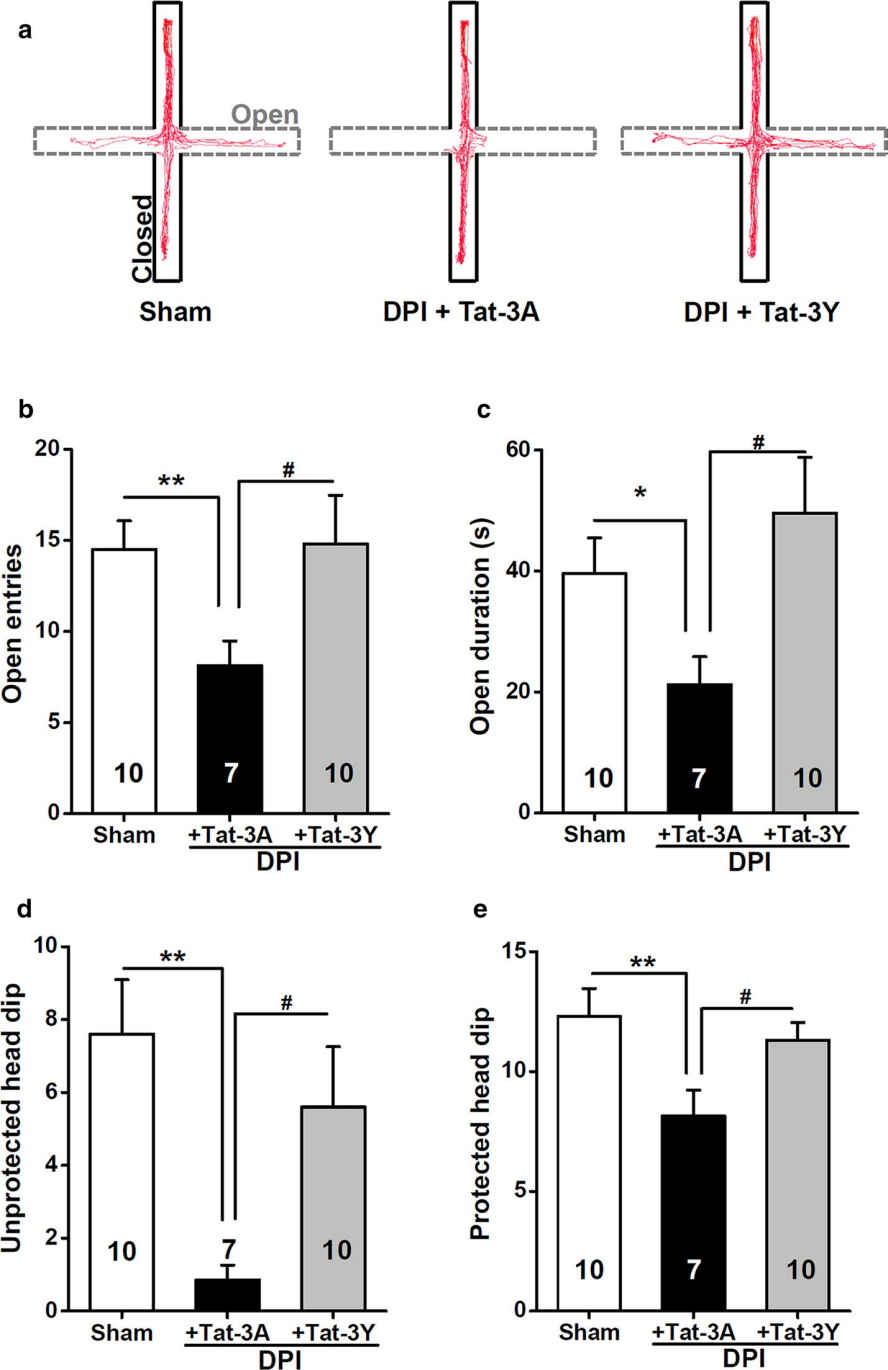
Effects of administration of Tat-3A or Tat-3Y on DPI-induced anxiety behaviors in the EPM 7 days after surgery. **a** Computer-generated exploration paths of representative mice in the EPM test subjected to the following conditions: sham control, DPI plus Tat-3A administration, DPI plus Tat-3Y administration. Open, *open arms* (*dashed line*, *grey*); closed, *closed arms* (*black*). **b**, **c** The *bar summary* shows the number of entries (**b**) and the time spent (**c**) in the *open arm*. **d**, **e** The *bar summary* shows the number of unprotected (**d**) and protected (**e**) head dips during the EPM test. All values are expressed as mean ± SEM. *n* = 7–10 mice for each group shown in the figure. *P < 0.05, **P < 0.01, sham vs. DPI + Tat-3A, unpaired Student’s *t* test. ^#^ *P* < 0.05, DPI + Tat-3A vs. DPI + Tat-3Y as indicated, unpaired Student’s *t* test.

Finally, we aimed to establish synaptic mechanisms that underlie DPI-induced anxiety. As mentioned above, a specific form of presynaptic LTP in ACC contributes to the interaction between anxiety and chronic pain [8]. By contrast, here we turned to examine whether the involvement of long-term depression (LTD) [35] act as a cellular mechanism to mediate anxiety phenotypes following DPI, as behaviorally stressful exposure facilitates LTD induction in hippocampus [36]. We anticipated that a particular depressed synaptic response [37] might confer the decreased exploring and increased anxiety observed in DPI compared to sham control mice. It is well known that endocytosis of α-amino-3-hydroxy-5-methyl-4-isoxazolepropionic acid receptor (AMPAR) acts a general mechanism of LTD [35] in the central nervous system, and that AMPAR endocytosis is dependent on GluA2 subunit and this process can be blocked by intracellular application of a peptide (GluA2-3Y) that mimics the C terminus of GluA2 [35, 38], but not by the mutant peptide (GluA2-3A). Mechanistically, the infusion peptide Tat-GluA2-3Y (Tat-3Y), designed via taking advantage of the delivery potential of the TAT peptide derived from HIV sequence [39], but not its control peptide Tat-GluA2-3A (Tat-3A), selectively blocked AMPAR endocytosis and various forms of LTD [35]. We then examined whether blockade of LTD could reverse DPI-induced anxiety. As expected, the systemic administration of Tat-3Y, but not Tat-3A, 1 h prior to behavioral tests on day 7 after DPI surgery, significantly reversed the resultant anxiety (Figure 10a). DPI mice treated with Tat-3Y significantly increased the number of entries (*P* < 0.01, sham vs. DPI + Tat-3A; *P* < 0.05, DPI + Tat-3A vs. DPI + Tat-3Y, Figure 10b) and time spent in the open arms of the maze (*P* < 0.05, sham vs. DPI + Tat-3A; *P* < 0.05, DPI + Tat-3A vs. DPI + Tat-3Y, Figure 10c) compared with that received the injection of Tat-3A. Moreover, Tat-3Y significantly increased the numbers of both unprotected (*P* < 0.01, sham vs. DPI + Tat-3A; *P* < 0.05, DPI + Tat-3A vs. DPI + Tat-3Y, Figure 10d) and protected (*P* < 0.01, sham vs. DPI + Tat-3A; *P* < 0.05, DPI + Tat-3A vs. DPI + Tat-3Y, Figure 10e) head dips during the EPM test, indicating that LTD blockade treatment indeed has an anxiolytic effect on DPI-induced anxiety. The present results support the major participation of an LTD mechanism in avoidance of exploring in DPI mice. In summary, based on the establishment of temporal dynamics between DPI and anxiety, we have further implicated a novel synaptic plasticity mechanism contributing to this communication.

## Discussion

Odontalgia is one of the most frequent reasons that patients seek stomatology care. Despite its prevalence in clinical practice as well as the strong likelihood that mood problems are concomitant with dental pain [10], few researchers have attempted mechanistic investigations [40] of odontalgia and the associated affective disorders using animal models, thus impeding the development of more efficacious therapeutic approaches. As a prototypical type of orofacial pain correlated with human toothache, the experimental DPI model [13–15] possesses multiple unique features that make it appropriate for use in neural and stomatologic studies. For instance, DPI can be specifically used to affect orofacial tissues, such as the teeth, tongue, and mucosa, without affecting other peripheral tissues. Because dental pulp lacks innervation by proprioceptive afferent fibers with larger diameters [14], and physiologic or pathologic stimulation of these tissues only evokes the sensation of pain, DPI represents an excellent animal model for studying affective comorbid disorders and neurophysiological mechanisms underlying odontalgia.

**Figure 11:**
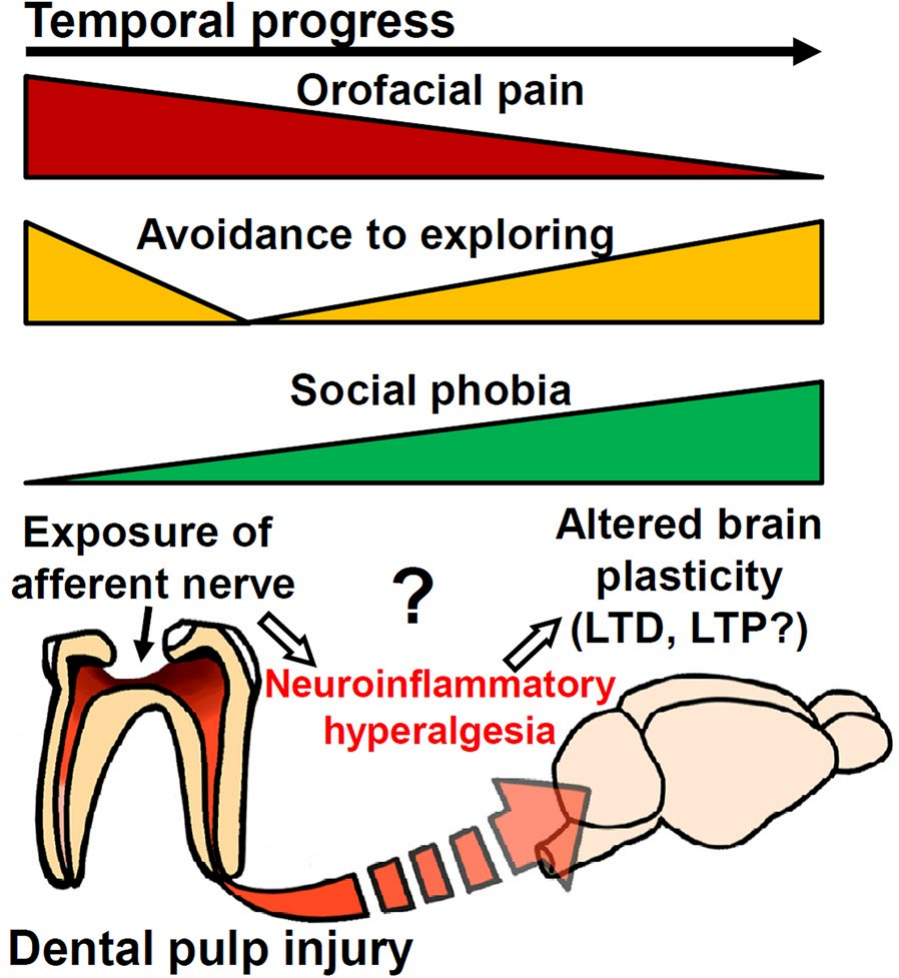
A hypothetical scheme for temporal dynamics of anxiety phenotypes in the dental pulp injury model. Please *see the text* for more details.

In the present study, we took advantage of the ease of generating DPI in mice [14]. We were thus able to comprehensively evaluate the associated behavioral and affective phenotypes at different time points following the DPI procedure. Based on our histological characterization of injured dental pulp and our behavioral evaluation of feeding- and nociception-related activities (Figures 1, 2), we concluded that DPI mice resembled a bell shape representing the appearance of anxious phenotypes over time (Figure 3). We further verified the anxiogenic phenotypes of DPI mice via ethological quantitation of behavior during EPM tests (Figure 4), and by additional analysis of behaviors during the open field (Figure 5) and NSF (Figures 6, 7) tests. DPI mice displayed avoidance to the open arm, center area, and novelty environment in the EPM, open field, and novelty-suppressed feeding tests, respectively. Strikingly, DPI was associated with social phobia (Figure 8) and increased repetitive grooming (Figure 4l) up to 14 days subsequent to the surgery, implying that the mice developed social phobia and obsessive–compulsive anxiety. Exploring the possible causes of DPI-induced anxiety, we found that both anti-inflammatory and analgesic treatments significantly relieved anxiety in DPI mice (Figure 9). Finally, to consider the central mechanisms of DPI-induced anxiety, we identified that pharmacological blockade of LTD significantly reduced the anxiety phenotypes subject to DPI (Figure 10). Taken together, our results contain new information about the temporal dynamics of anxiety emergence in an orofacial pain model (Figure 11), in addition to shed more lights on the underlying mechanisms, thus providing a primary basis for further in-depth studies regarding the mechanisms of comorbid pain and anxiety disorders [2–4].

The neural mechanisms underlying here identified temporal dynamics of anxiogenic emergence subsequent to DPI remain unclear. There are several possibilities that could be considered in future research. The temporal dynamics of the appearance of various anxiogenic phenotypes in the DPI model might partially correlate with the pathological development of chronic orofacial hyperalgesia [11, 12, 14, 15]. As described previously [12, 14], mechanical exposure of the dental pulp induces inflammatory changes in the pulp as early as 1 day after surgery while periradicular changes occur at least 5 days after the surgery. Considering the fact that anxious phenotypes did not occur 3 days after DPI surgery, we speculated that the injury *per se* together with inflammation (Figure 1) and related pain (Figure 2) were not the only determinants of anxiogenic effects of DPI. Alternatively, we preferred that sustained rather than transient pain might be more critical for the anxiogenic effects. For the DPI in our animal model, an increased pain-like behavior continued for at least a week (Figure 2). Consistently, except 1 day after DPI, we did not observe increases in avoidance to the open arm, center area, or novelty environment in the EPM, open field, and NSF tests, respectively, until 1 week after DPI procedure. Moreover, we did not observe social phobia and increased repetitive grooming in DPI mice until at least 2 weeks after the surgery. It is worth noting that a previous study showed that in a neuropathic pain model of sciatic nerve ligation [41], when hyperalgesia was reversed, the anxiogenic effect was lost. However, in our experiment, the anxiogenic effects of DPI still persisted in spite of disappearance of pain-like behavior compared to the sham control. Nevertheless, these data collectively direct a notion that sustained hyperalgesia necessitates the initiation and expression of anxious phenotypes. Overall, we believed that the temporally-dependent anxiogenic effects of DPI (Figure 11) identified here raise an important view regarding affective comorbidity [10, 11] in the field of stomatology.

The neural mechanisms mediating affective disorders concomitant with DPI remain unexplored. Although the anxiolytic effects of either anti-inflammatory or analgesic treatment on DPI provided important information about the mechanisms of orofacial pain, the neural pathways, from peripheral sensory sensitization to the modification of specific brain regions, remain elusive. At the peripheral level, nociceptor excitability modulation via multiple molecular and cellular mechanisms [42], such as the regulation of emerging ion channel targets [43, 44], will represent the first step in mediating the sensory, affective, and even cognitive components [45] of persistent pain certainly including DPI. At the central level, two contrasting forms of LTP in ACC are required for the anxiogenic effects of inflammatory and neuropathic pain to take place [8]. In the current study, we added the central LTD to the list of cellular mechanisms which underlie the DPI-caused anxiety (Figure 11), although the precise site of this form of synaptic adaptation together with the molecular details remain elusive, which need to be clarified in the future studies. Except ACC [8], it has been increasingly considered that adaptive changes occurred in additional brain regions such as amygdala [41], dentate gyrus [9], hippocampus and prefrontal cortex [46] responsible for the interaction between sensory and affective components. Mechanistically, comprehensive deciphering synaptic adaption mechanisms in these regions under chronic pain are essentially required towards the understanding on the complex relationship between pain and anxiety. Of note, altered amygdala opioidergic function [41, 47, 48] has been identified to play significant roles in modification of reward and anxiety states associated with chronic pain. Consistently, DPI mice exhibit a paradoxical increase of sucrose consumption [13], a behavior index likely involving a convergence of neuronal pathways that underlie pain and reward. Not only, here we characterize an increased anxiety in DPI mice, a phenotype probably mediated by central plasticity interplaying with pain and anxiety [49]. Moreover, a recent study demonstrated a decreased motivation during chronic pain [37] that was associated with LTD in the nucleus accumbens. Overall, on the basis of these progresses, future studies are indeed required to make out the complicated relationship between pain-associated ethological changes and the respective adaptive plasticity. We believe that our data no doubt assist in achieving this goal by establishing the temporal dynamics of anxiety emergence in DPI.

## Methods

### Animals

Animal care and all procedures were approved by the Animal Ethics Committee of Shanghai Jiao Tong University School of Medicine, Shanghai, China. The male C57BL/6J mice (2–3 months old) used in the present study were obtained from Shanghai Slac Laboratory Animal Company Limited (Shanghai, China). The animals were housed three to four animals per cage and maintained on a 12 h light/dark cycle with food and water ad libitum excepted where otherwise indicated. Animals were acclimatized to the testing room for at least 1 h before all behavioral experiments, and we conducted each behavioral assessment once for each animal, in a randomized and blind order.

### Dental pulp injury and sham surgeries

We conducted DPI surgeries, in which we mechanically exposed the dental pulp, and sham control surgeries on mice, as previously reported [13, 19, 40]. Under sodium pentobarbital-induced anesthesia [intraperitoneally (i.p.), 10 mg/kg], the mouth of the mouse was opened and the tongue gently retracted using forceps. Next, the dental pulp of left maxillary first molar was exposed by means of a low-speed dental drill with a 1/4 round tungsten carbide bur powered by a variable-speed electric rotary hand piece. The exposures were confirmed using a 6K-file (Mani Inc., Japan) and the procedure was illuminated via a surgical microscope. Animals with accidental damage supplementary to the intended pulp exposure were excluded from analysis. Sham control animals received identical anesthesia, and their mouths were kept open with forceps for approximately 5 min, similar to the time required to complete the DPI procedure. All animals were returned to their home cages after recovery from anesthesia, with free access to food and water.

### Histological evaluation

We used a procedure similar to that previously described [13] to verify the success of the operation. In brief, mice were anesthetized with an i.p. application of sodium pentobarbital and transcardially perfused with isotonic saline followed by a fixative containing 4% paraformaldehyde in 0.1 M phosphate buffer (pH 7.4). The entire maxilla was collected and post-fixed in the same fixative for 48 h at 4°C. The tissue was then cryoprotected overnight in 30% sucrose in 0.1 M phosphate buffer. After decalcifying the samples in 10% ethylenediaminetetraacetic acid (pH 7.6) for 4–5 weeks, the tissue was sectioned at 5 μm and thaw-mounted onto Superfrost Plus microscope slides (Fisher Scientific, Pittsburgh, PA, USA). The slides underwent a standard hematoxylin and eosin (H&E) staining protocol for visualization of gross histological structures.

### Feeding, drinking, and body weight

After the mice had fully adapted to the rearing environment, we measured their food intake, water consumption, and body weight once each day for 5–7 continuous preoperative days and 7 continuous postoperative days. We used the average bodyweight values from the last 3 days and the average water and food intake measures from the last 2 days before the surgery as the preoperative baselines, as shown in Figure 1c–e. The mice had free access to food, which consisted of standard rodent food pellets. The mice also had free access to pipettes containing over 50 ml of water, which was replaced with fresh daily. Water pipettes, food pellets, and body weights were measured using an electronic scale.

### Nociceptive assessment

Nociceptive behaviors indexed by spontaneous face grooming were evaluated as described previously [12, 15]. Briefly, mice subjected to sham control or DPI surgery were first placed in a Plexiglas apparatus (10 × 10 × 14 cm) in a dimly lit room, after a 30-min adaption period, and were videotaped for another 30 min. The frequency and duration that the mice spent in rubbing the ipsilateral face with left fore- or hind-paw during the last 30 min were counted by an observer who was blind to the surgery treatment.

### Elevated plus maze test

To investigate changes in anxiogenic effects, we subjected the mice to the elevated plus-maze (EPM) test, which has been used extensively to identify novel anxiolytic agents and to investigate the physiological and neurochemical bases of anxiety [21–23]. The EPM was made of grey acryl glass and elevated at a height of 50 cm above the floor. It consisted of four equally spaced arms (30 × 6 cm) radiating out from a central area (6 × 6 cm). Two opposing closed arms were enclosed from all sides except for the side adjoining the central area, and the remaining two open arms were exposed. A digital camera was mounted above the maze to record the images, which were quantified using the Ethovision video tracking system (Noldus Information Technology, Wageningen, Netherlands). Trials, which lasted 5 min, began once an animal was placed gently in the center area, facing one of the open arms. After each trial, the entire maze was cleaned and animal was returned to the home cage.

### Ethological measurements during the EPM test

We performed further ethological analysis during the EPM test to identify innate anxiety behaviors in mice. Our methods were modified from previous reports [24, 50, 51]. The frequencies of the following behaviors were recorded and summarized. Head dipping, which was defined as instances in which the animal stuck its head outside the maze border and below the level of the maze floor, was further categorized into unprotected and protected types based on whether the behavior occurred on the open arms or the center area and closed arms of the maze, respectively. Rearing was characterized as instances in which the animal stood on its hind limbs or leaned against the maze walls using its front paws. Finally, grooming was defined as instances in which the animal licked or scratched itself using its paws and/or mouth while stationary.

### Open field test

The open field test [52] is another behavioral assay that has been widely used to evaluate innate anxiety-like behaviors and locomotor responses to novel environments in rodents [23, 53, 54]. We conducted the open field test in a square Plexiglas apparatus (40 × 40 × 35 cm) under diffused lighting [55]. In detail, the arena was partitioned such that there was a “center” zone (20 × 20 cm) and a “corner” zone occupying the remaining area [26]. A digital camera was set directly above the apparatus. Images were captured at a rate of 5 Hz and quantified using the Ethovision video tracking system (Noldus Information Technology). Mice were gently placed in the center of the square and allowed to explore freely for 5 min. After each trial, the apparatus was cleaned and the animal returned to the home cage.

### Novelty-suppressed feeding test

The novelty-suppressed feeding test has also been validated as a test that is sensitive to anxiety-related behavior [27, 28]. The novelty-suppressed feeding test apparatus consisted of a plastic arena (38 × 32 × 16 cm) filled with wood-chip bedding material at a depth of approximately 2 cm. A single food pellet was placed on a piece of white filter paper on a food platform (9 cm in diameter) positioned in the center of the arena. Mice were deprived of food in their home cages for 24 h before test. The test began when a mouse was gently placed in a random corner of the arena. We recorded the amount of time that passed before the mouse approached the pellet and began feeding, to a maximum of 10 min. After 10 min, the mouse was immediately transferred to the home cage and we measured the amount of food consumed there within 5 min. We measured the weight of each mouse before food deprivation, before testing, and after testing.

### Social exploration test

We conducted the social exploration test in a similar manner to that previously described [29, 55]. Each mouse was placed in a new cage and a naïve male C57BL/6J mouse (3 weeks old) was introduced. The two mice were left to explore freely for 5 min. A digital camera was set directly above the cage and the interactions between the mice were recorded. An observer who was blind to the surgery treatment timed the exploratory behaviors initiated by the adult mouse towards the juvenile, including sniffing, pinning, and allogrooming.

### Drug administration

Excepted where otherwise indicated, all drugs were purchased from Sigma-Aldrich (St. Louis, MO, USA). Freshly-prepared drugs were administered in drinking water that was freely accessible to the animals. Ibuprofen was dissolved in water with minimum hydrotropic agent PEG/DMSO (polyethylene glycol/dimethyl sulfoxide, 50%) and administered at a concentration of 0.2 mg/ml, which has been suggested to be effective [31] in mice. ProTx-II was added to drinking water at a concentration of 0.5 μg/ml [32]. TAT-fusion peptides (TAT-GluA2-3Y peptide, “Tat-3Y”: YGRKKRRQRRR-YKEGYNVYG, GL Biochem Ltd, Shanghai, China) containing the TAT (YGRKKRRQRRR) sequence, a trans-activating domain of HIV protein that can permeate the cell membrane [39], and its mutant control-peptide (TAT-GluA2-3A peptide, “Tat-3A”: YGRKKRRQRRR-AKEGANVAG) were administered intraperitoneally (i.p.) (5 μmol in 10 ml saline per kg mice). The mass and purity of the peptides were verified by high-performance liquid chromatography. Peptides were freshly prepared in saline solution and injected at 500 μM in mice.

### Data analysis

Water intake (Figure 1c), food intake (Figure 1d), and body weights (Figure 1e) were analyzed using a two-way (manipulation × days) analysis of variance (ANOVA). The Bonferroni corrected post hoc t tests alongside ANOVA were performed. All data obtained in the pain-like behaviors (Figure 2), EPM (Figures 3, 4, 9, 10), open field (Figure 5), and social exploration (Figure 8) tests were analyzed using Student’s *t* tests (two tailed). The data obtained in the novelty-suppressed feeding test were first subjected to Student’s *t* tests (two tailed) (Figures 6b, d, e, g, 7a, c, d, f), and then to cumulative distribution analyses (Figures 6c, f, 7b, e), performed using a one-sample Kolmogorov–Smirnov test. All results are expressed as the mean ± SEM. Except where noted otherwise, *P < 0.05, **P < 0.01, and ***P < 0.001 represent significant differences.

## Abbreviations

AMPAR: α-amino-3-hydroxy-5-methyl-4-isoxazolepropionic acid receptor
ANOVA: analysis of variance
DMSO: dimethyl sulfoxide
DPI: dental pulp injury
EPM: elevated plus maze
H&E: hematoxylin and eosin
i.p.: intraperitoneally
LTD: long-term depression
LTP: long-term potentiation
NSF: novelty-suppressed feeding
PEG: polyethylene glycol
TRPA1: transient receptor potential cation channel, subfamily A, member 1
