# Erratum to: Temporal dynamics of anxiety phenotypes in a dental pulp injury model

**DOI:** 10.1186/s12990-015-0064-8

**Published:** 2015-10-07

**Authors:** Lin Shang, Tian-Le Xu, Fei Li, Jiansheng Su, Wei-Guang Li

**Affiliations:** Laboratory of Oral Biomedical Science and Translational Medicine, School of Stomatology, Tongji University, Shanghai, 200072 China; Department of Developmental and Behavioral Pediatrics, Shanghai Institute of Pediatric Translational Medicine, Shanghai Children’s Medical Center, Ministry of Education-Shanghai Key Laboratory of Children’s Environmental Health, Shanghai Jiao Tong University School of Medicine, Shanghai, 200129 China; Discipline of Neuroscience and Department of Anatomy, Histology and Embryology, Institute of Medical Sciences, Shanghai Jiao Tong University School of Medicine, 280 South Chongqing Road, Shanghai, 200025 China

## Erratum to: Mol Pain (2015) 11:40 DOI 10.1186/s12990-015-0040-3

Following the publication of our original article we noticed that an error was introduced during the assembly of Fig. 3a (Fig. 1a here). In the original figure, the computer-generated exploration path of DPI mice at day 1 was incorrectly presented, as we mistakenly included a duplication of the exploration path representing day 14. The corrected figure appears below.

**Fig. 1:**
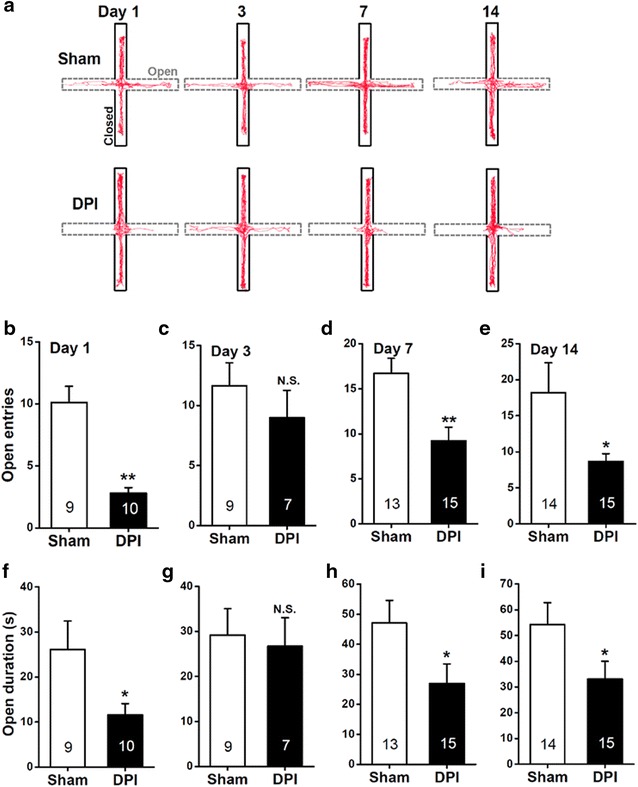
Measurements of anxiety-like behavior in the EPM after sham or DPI surgery. **a** Computer-generated exploration paths of representative sham and DPI mice in the EPM test. Open, *open arms* (*dashed line*, *grey*); closed, *closed arms* (*black*). **b**–**i** The bar summary compares the number of entries (**b**–**e**) and the amount of time spent (**f**–**i**) in the *open arms* between the sham and DPI mice. All values are expressed as mean ± SEM. *n* = 7–15 mice for each group shown in the figure. *P < 0.05, **P < 0.01, *N.S.* non-significant difference, sham vs. DPI, unpaired Student’s *t* test

